# Mapping the PHQ-8 to EQ-5D, HUI3 and SF6D in patients with depression

**DOI:** 10.1186/s12888-021-03463-0

**Published:** 2021-09-13

**Authors:** Edimansyah Abdin, Siow Ann Chong, Esmond Seow, Kelvin Bryan Tan, Mythily Subramaniam

**Affiliations:** 1 Research Division, Institute of Mental Health, 10 Buangkok Viewm, Singapore, 539747 Singapore; 2 Ministry of Health, Singapore, Singapore; 3 Saw Swee Hock School of Public Health, National University of Singapore, Singapore, Singapore

## Abstract

**Background:**

There is limited evidence of mapping clinical instruments to a generic preference-based instrument in Asian patient populations. The current study aims to map the eight-item Patient Health Questionnaire depression scale (PHQ-8) onto the EuroQol Five-Dimension (EQ-5D), the Health Utilities Index Mark 3 (HUI3) and the Short Form Six-Dimension (SF-6D) which helps to inform future cost-utility analyses of treatments for depression.

**Methods:**

A total of 249 participants who had completed PHQ-8, EQ-5D, SF-6D and HUI3 questionnaires were included in the analyses. A beta regression mixture model was used to map the utility scores as a function of PHQ-8 total scores, PHQ-squared, age and gender. The predictive accuracy of the models was examined using mean absolute error and root mean square error.

**Results:**

The results were compared against two common regression methods including Ordinary Least Square (OLS) and Tobit regression models. The mean age of the sample was 36.2 years (SD = 11.1). The mean EQ-5D-3L, EQ-5D-5L, HUI3 and SF-6D utility scores were 0.615, 0.709, 0.461 and 0.607, respectively. The EQ-5D-3L, EQ-5D-5L and SF-6D utility scores were best predicted by the beta mixture regression model consisting of PHQ-8 total sores, PHQ-squared, and covariates including age and gender. The HUI3 was best predicted by the OLS regression model.

**Conclusions:**

The current study provides important evidence to clinicians and researchers about the mapping algorithms that can be used in economic evaluation among patients with depression.

**Supplementary Information:**

The online version contains supplementary material available at 10.1186/s12888-021-03463-0.

## Introduction

Depression is a severe mental disorder that causes substantial impairment to the individual and a significant burden to their family members and society. It is a highly prevalent mental disorder affecting 264 million of the global population. The total direct excess costs of depression per person ranges from USD$124 to USD$18,174 in adults and between $2868 and $2883 in adolescents [1]. The cost of lost productivity in terms of absenteeism and presenteeism varies across countries. The absenteeism costs associated with depression were the highest in Japan ($2674), while presenteeism costs were $5524 in the United States and $5788 in Brazil [2]. Depression has been strongly linked to an increased risk of suicide which is the leading cause of death among adolescents [3]. Due to increasing efforts worldwide to develop more effective treatment options and strategies for people with depression, there is a growing need for conducting health technology assessments such as cost-effectiveness analysis (CEA) and cost-utility analysis (CUA) to assess the quality, safety, efficacy, and cost-effectiveness of services.

The EuroQol Five-Dimension (EQ-5D), Short Form Six-Dimension (SF-6D), and Health Utilities Index Mark 3 (HUI3) are commonly used generic preference-based instruments to measure health-related quality of life among patients with depression in the literature [4, 5]. These instruments are used to calculate quality-adjusted life-years (QALYs) in CEA and CUA. In the clinical setting, however, these instruments are often not used. Therefore, mapping a clinical instrument to a generic preference-based instrument to generate statistical formulas or functions that allow the clinical instruments to estimate utility scores provides an alternative solution to generate QALYs for CEA and CUA in clinical studies [5, 6]. The Patient Health Questionnaire (PHQ) [7] is one of the most widely used clinical instruments to measure symptom severity of depression in a clinical setting. Hence, developing a mapping function based on PHQ that can produce accurate utility scores would help clinicians and psychiatrists address the unmet needs for CEA and CUA among patients with depression. These mapping functions are particularly useful when comparing QALYs results of patients with depression across treatments, interventions, and care programs. Clinicians and psychiatrists from public hospitals as well as policymakers would find this helpful in identifying needs when planning healthcare services, setting priorities, allocating resources, and evaluating outcomes and effectiveness of the treatments, interventions, and care programs in the clinical setting and community. Given that there is limited data on mapping studies using the PHQ to estimate the utility scores among people with depression, the current study aims to map the PHQ onto the EQ-5D, HUI3, and SF-6D to inform cost-utility analyses of treatment for depression.

## Methods

The study was conducted between August 2016 and November 2017 at a tertiary psychiatric hospital, which serves the majority of psychiatric patients in Singapore. Patients were included in the study if they were Singapore citizens or permanent residents, aged 21 years and above, literate in English, and had a clinical diagnosis of depressive disorder. A total of 249 participants who had completed PHQ-8, EQ-5D, SF-6D and HUI3 questionnaires were included in the analyses.

The relevant institutional ethics review board approved the study (National Healthcare Group Domain Specific Review Board (DSRB) (Reference no: 2016/00215). Written informed consent was obtained from all study participants.

### Measures

#### PHQ-8

The eight-item Patient Health Questionnaire (PHQ-8) is a self-reported questionnaire designed to measure depressive symptom severity in research and clinical care [7]. It assesses how often in the past 2 weeks, participants experienced eight depressive symptoms. Each symptom is rated on a 4-point Likert scale ranging from 0 (not at all) to 3 (nearly every day), with total scores ranging from 0 to 24. The PHQ-8 has been widely used to measure the severity of depressive symptoms in psychiatric patients in Singapore [8, 9].

#### EQ-5D

The EQ-5D is a generic preference-based measure for subjectively describing and valuing health-related quality of life that has been developed by the EuroQol Group [10]. It comprises two versions – EQ-5D-3L and EQ-5D-5L. The EQ-5D-3L includes five questions on mobility, self-care, pain, usual activities, and psychological status with three possible answers for each item (1 = no problem, 2 = moderate problem, 3 = severe problem). The utility scores of EQ-5D-3L were calculated using the scoring algorithm developed in Singapore [11]. The EQ-5D-5L is a new version of the EQ-5D comprising five questions on mobility, self-care, pain, usual activities, and psychological status with five possible responses for each item (1 = no problem, 2 = slight problems, 3 = moderate problems, 4 = severe problems, 5 = extreme problems). The utility scores of EQ-5D-5L were developed by van Hout et al. using a crosswalk project that maps EQ-5D-5L utility scores from the EQ-5D-3L [12].

#### HUI3

The HUI3 is a generic comprehensive health status classification instrument [13]. It generates utility scores using a utility scoring function derived from a representative sample of the general Canadian population based on the Standard Gamble and visual analogue scale methods [14]. The utility score ranged between − 0.36 and 1. The HUI3 comprises eight domains: vision, hearing, speech, ambulation, dexterity, emotion, cognition, and pain. Per attribute, 5 to 6 levels are derived from 15-multiple choice questions. The utility scores obtained from Chinese and Malay versions of the HUI3 have been found to be equivalent and valid in Singapore [15].

#### SF-6D

The Short Form-36 Health Survey is a generic instrument that can be used to generate SF-6D utility scores using a utility scoring function derived from a representative sample of the general UK population [16]. The utility score ranged between 0.29 and 1. It has six domains: physical functioning, role limitation, social functioning, pain, mental health, and vitality, with 4–6 levels for each domain. The utility scores derived from Chinese and English versions of the SF-6D have been demonstrated to be equivalent and valid in Singapore [17].

### Statistical analyses

Statistical analyses were carried out using the STATA software version 13 (StataCorp LP, College Station, TX). The overlap between the source and the target instruments were calculated using the Spearman correlation coefficient. Since the distribution of utility scores derived from generic preference-based measures such as EQ-5D are often not normally distributed and have a higher ceiling effect at a value of 1 [18], we used a beta regression mixture model (betamix) to map the utility scores. In this study, a beta mixture regression model was used as a primary statistical model to predict different points of health instruments. The model has an advantage over other regression models in terms of its flexibility and ability to capture different points of health utility scores, including negative values (health state worse than death), the peak of observation at full health or death, the gap between boundary values and a mixture of number components of beta distributions. The results were compared against two common regression methods, including Ordinary Least Square (OLS) and Tobit regression models [19]. The beta regression mixture model is a two-part model that incorporates a multinomial logit model and a beta mixture model in their algorithms. Studies have increasingly suggested that this regression method outperforms the linear regression model [20–22]. In order to determine the best performance of the prediction model, three regression methods with 18 different model specifications consisting of three model specifications in each OLS and Tobit, and 12 model specifications in the beta mixture regression models with up to two components with and without truncation and probability mass at full health and truncation point were included in the current analyses. The first model included only PHQ-8 total scores as a main predictor for the utility score; the second model included PHQ-8 total scores, age, and gender. The third model included PHQ-8 total scores, PHQ-squared, age, and gender. The performance of regression methods was assessed using the following criteria. Both mean absolute error (MAE) and root mean square error (RMSE) were used as the main criterion to compare the performance of regression methods. Values from both indices were ranked and summed to get an average ranking. The regression model with the lowest average ranking values (ARV) was considered to be the best prediction model [6, 22, 23].

## Results

### Descriptive statistics

**Fig. 1:**
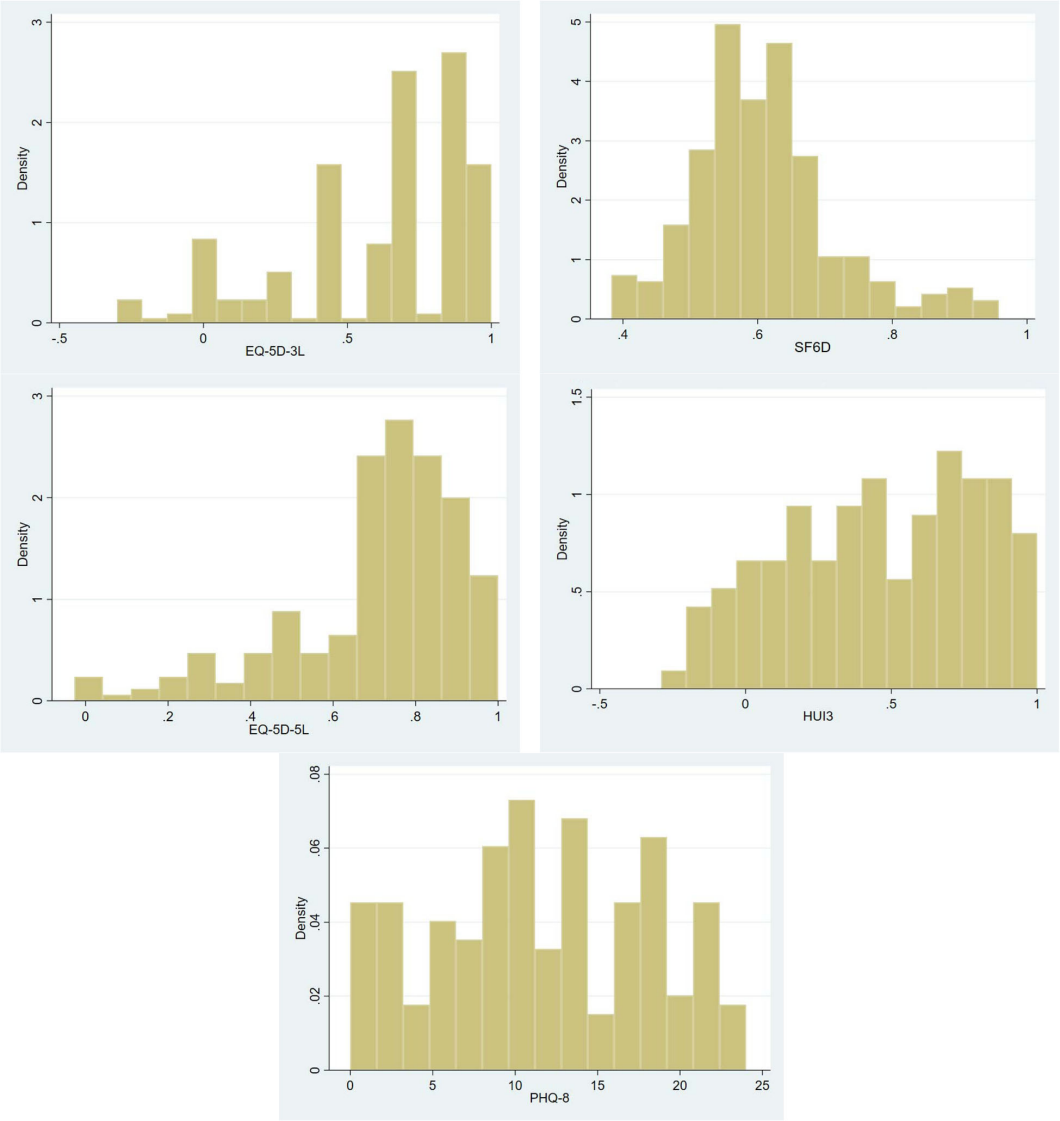
Distribution of EQ-5D-3L, EQ-5D-5L, HUI3, SF-6D and PHQ-8 in depression sample

The descriptive statistics are presented in Table 1. The sample included 249 participants with depression. The mean age of the overall sample was 36.2 years (SD = 11.1), 69.9% were Chinese, 13.7% were Malays, 14.4% were Indians, and 2% belonged to other ethnicities. The EQ-5D-3L showed a mean (SD) index score of 0.615 (0.317) with minimum and maximum scores of − 0.2999 and 1, while the mean (SD) EQ-5D-5L index was 0.709 (0.212) with minimum and maximum scores of − 0.027 and 1, respectively. The mean (SD) HUI3 index score was 0.461 (0.331) with minimum and maximum scores of − 0.289 and 1, while the mean SF-6D was 0.607 (0.105) with minimum and maximum scores of 0.385 and 0.958, respectively. The distribution of the EQ-5D-3L, EQ-5D-5L, and HUI3 utilities showed a substantial skew to the right, that is, toward a better quality of life (Fig. 1). The mean (SD) PHQ-8 total score was 11.526 (6.590), with minimum and maximum scores of 0 and 24, respectively.

**Table 1:** Characteristics of the sample

	N (%)
Demographic profiles	
**Age**, Mean (SD)	36.2 (11.1)
**Gender**	
Female	118 (47.4)
Male	131 (52.6)
**Ethnicity**	
Chinese	174 (69.9)
Malay	34 (13.7)
Indian	36 (14.4)
Others	5 (2.0)
Utilities	
EQ-5D-3L, Mean (SD)	0.615 (0.317)
EQ-5D-5L, Mean (SD)	0.709 (0.212)
HUI3, Mean (SD)	0.461 (0.331)
SF-6D, Mean (SD)	0.607 (0.105)
**PHQ-8 total scores, Mean (SD)**	**11.526(6.590)**

### Correlations between source and target instruments

Table 2 shows the Spearman ‘s correlation coefficient results between the source and the target instruments. A strong inverse correlation was observed between the source instrument, i.e., PHQ-8, and the four target instruments (EQ-5D-3L, EQ-5D-5L, HUI3, and SF-6D). The correlation coefficient ranged between -0.61 and -0.78, suggesting a significant overlap between the source and the target instruments.

**Table 2:** Pearson’s correlation coefficients between PHQ-8, EQ-5D-3L, EQ-5D-5L, HUI3 and SF-6D

Utilities	PHQ-8
**EQ-5D-3L**	**−0.664**
**EQ-5D-5L**	**−0.614**
**HUI3**	**−0.783**
**SF-6D**	**−0.692**

### Mapping on EQ-5D-3L

Table 3 shows the performance of three regression methods (beta mixture regression, OLS, and Tobit) for mapping PHQ-8 to the EQ-5D-3L utility scores. Among the three regression methods and 18 model specifications, beta mixture regression method with two components with truncation, probability mass at full health (1), and the truncation point (0.8538) was the most parsimonious prediction model for the EQ-5D-3L utility scores. It produced the smallest average ranking of MAE (0.1765) and RMSE (0.2326) values compared to other regression methods and model specifications (Table 3). This regression model revealed that PHQ-squared scores were significantly and negatively associated with EQ-5D-3L utility scores in the first component and found that the PHQ-8 total scores and PHQ-squared were negatively and positively associated with the full health (Supplementary Table 1).

**Table 3:** Model performance of three regression methods for mapping the PHQ-8 to the EQ-5D-3L utility scores

No	Mapping method	Number of components and truncation	Specification	ME	MAE	RMSE	MAE rank	RMSE rank	ARV
1	BETAMIX M1a	1 component without truncation	Probability mass at full health	0.0904	0.2014	0.2621	18	18	18
2	BETAMIX M1b	2 components without truncation	Probability mass at full health	0.0130	0.1868	0.2381	15	11	13
3	BETAMIX M1c	2 components with truncation	Probability mass at full health	−0.0043	0.1839	0.2370	12	9	10.5
4	BETAMIX M1d	2 components with truncation	Probability mass at full health and truncation point	−0.0024	0.1861	0.2390	13	13	13
5	BETAMIX M2a	1 component without truncation	Probability mass at full health	0.0866	0.1962	0.2607	17	17	17
6	BETAMIX M2b	2 components without truncation	Probability mass at full health	0.0101	0.1825	0.2349	8	7	7.5
7	BETAMIX M2c	2 components with truncation	Probability mass at full health	−0.0038	0.1806	0.2341	6	5	5.5
8	BETAMIX M2d	2 components with truncation	Probability mass at full health and truncation point	−0.0013	0.1813	0.2355	7	8	7.5
9	BETAMIX M3a	1 component without truncation	Probability mass at full health	0.0659	0.1864	0.2504	14	16	15
10	BETAMIX M3b	2 components without truncation	Probability mass at full health	0.0119	0.1800	0.2321	5	1	3
11	BETAMIX M3c	2 components with truncation	Probability mass at full health	0.0020	0.1774	0.2328	2	3	2.5
12	BETAMIX M3d	2 components with truncation	Probability mass at full health and truncation point	**0.0057**	**0.1765**	**0.2326**	1	2	1.5
13	OLS M1			0.0000	0.1837	0.2374	11	10	10.5
14	OLS M2			0.0000	0.1798	0.2347	4	6	5
15	OLS M3			0.0000	0.1784	0.2331	3	4	3.5
16	TOBIT M1			−0.0263	0.1870	0.2413	16	15	15.5
17	TOBIT M2			−0.0264	0.1834	0.2389	9	12	10.5
18	TOBIT M3			−0.0264	0.1836	0.2390	10	14	12

*NOTE*: *ME* Mean error, *MAE* Mean absolute error, *RMSE* Root mean square error, *ARV* Average ranking values

M1 = Regression model including PHQ as explanatory variable

M2 = Regression model including PHQ, age, gender as explanatory variables

M2 = Regression model including PHQ, PHQ-squared, age, gender as explanatory variables

### Mapping on EQ-5D-5L

Table 4 shows the performance of three regression methods for mapping the PHQ-8 to the EQ-5D-5L utility scores. Among the three regression methods and 18 model specifications, beta mixture regression method with two components with truncation and probability mass at full health (1) and truncation point (0.879) was the most parsimonious prediction model for the EQ-5D-5L utility scores. It produced the best prediction performance index (MAE = 0.1208 and RMSE = 0.1620) than other regression methods and other model specifications. In this regression model (Supplementary Table 2), age was significantly and negatively associated with the EQ-5D-5L utility scores in the first component utility scores. In contrast, PHQ-squared was significantly and negatively associated with the EQ-5D-5L utility scores in the second component and PHQ-squared and age were significantly and negatively associated with the full health (Supplementary Table 2).

**Table 4:** Model performance of three regression methods for mapping the PHQ-8 to the EQ-5D-5L utility scores

No	Mapping method	Number of components and truncation	Specification	ME	MAE	RMSE	MAE rank	RMSE rank	ARV
1	BETAMIX M1a	1 component without truncation	Probability mass at full health	0.0354	0.1374	0.1720	18	18	18
2	BETAMIX M1b	2 components without truncation	Probability mass at full health	0.0010	0.1296	0.1708	15	17	16
3	BETAMIX M1c	2 components with truncation	Probability mass at full health	−0.0007	0.1274	0.1687	11	14	12.5
4	BETAMIX M1d	2 components with truncation	Probability mass at full health and truncation point	0.0031	0.1293	0.1696	14	16	15
5	BETAMIX M2a	1 component without truncation	Probability mass at full health	0.0355	0.1338	0.1691	17	15	16
6	BETAMIX M2b	2 components without truncation	Probability mass at full health	−0.0018	0.1254	0.1670	7	11	9
7	BETAMIX M2c	2 components with truncation	Probability mass at full health	0.0002	0.1243	0.1656	5	8	6.5
8	BETAMIX M2d	2 components with truncation	Probability mass at full health and truncation point	0.0051	0.1258	0.1657	10	9	9.5
9	BETAMIX M3a	1 component without truncation	Probability mass at full health	0.0357	0.1297	0.1663	16	10	13
10	BETAMIX M3b	2 components without truncation	Probability mass at full health	−0.0011	0.1213	0.1631	3	2	2.5
11	BETAMIX M3c	2 components with truncation	Probability mass at full health	0.0031	0.1212	0.1632	2	4	3
12	BETAMIX M3d	2 components with truncation	Probability mass at full health and truncation point	**0.0023**	**0.1208**	**0.1620**	1	1	1
13	OLS M1			0.0000	0.1279	0.1673	13	12	12.5
14	OLS M2			0.0000	0.1255	0.1642	8	5	6.5
15	OLS M3			0.0000	0.1238	0.1631	4	3	3.5
16	TOBIT M1			−0.0098	0.1278	0.1681	12	13	12.5
17	TOBIT M2			−0.0101	0.1256	0.1653	9	7	8
18	TOBIT M3			−0.0098	0.1245	0.1644	6	6	6

### Mapping on HUI3

Table 5 shows the performance of three regression methods for mapping the PHQ-8 to the HUI3 utility scores. Among the three regression methods and 18 model specifications, the OLS regression method with model 3 specification performed as the most parsimonious prediction model for the HUI3 utility scores. It produced the best prediction performance index (MAE = 0.1584 and RMSE = 0.2024). In this regression model, those with lower PHQ-8 total scores and of younger age were significantly associated with higher HUI3 scores (Supplementary Table 3).

**Table 5:** Model performance of three regression methods for mapping the PHQ-8 to the HUI3 utility scores

No	Mapping method	Number of components and truncation	Specification	ME	MAE	RMSE	MAE rank	RMSE rank	ARV
1	BETAMIX M1a	1 component without truncation	Probability mass at full health	−0.2680	0.2844	0.3644	17	17	17
2	BETAMIX M1b	2 components without truncation	Probability mass at full health	−0.0013	0.1664	0.2077	13	13	13
3	BETAMIX M1c	2 components with truncation	Probability mass at full health	−0.0001	0.1666	0.2082	14	14	14
4	BETAMIX M1d	2 components with truncation	Probability mass at full health and truncation point	−0.0007	0.1662	0.2074	12	12	12
5	BETAMIX M2a	1 component without truncation	Probability mass at full health	−0.2682	0.2842	0.3624	16	16	16
6	BETAMIX M2b	2 components without truncation	Probability mass at full health	.	.	.			
7	BETAMIX M2c	2 components with truncation	Probability mass at full health	−0.0010	0.1607	0.2023	7	1	4
8	BETAMIX M2d	2 components with truncation	Probability mass at full health and truncation point	0.0002	0.1606	0.2025	6	6	6
9	BETAMIX M3a	1 component without truncation	Probability mass at full health	−0.2679	0.2839	0.3623	15	15	15
10	BETAMIX M3b	2 components without truncation	Probability mass at full health	−0.0048	0.1602	0.2027	5	7	6
11	BETAMIX M3c	2 components with truncation	Probability mass at full health	−0.0026	0.1612	0.2028	8	8	8
12	BETAMIX M3d	2 components with truncation	Probability mass at full health and truncation point	−0.0158	0.1634	0.2048	11	9	10
13	OLS M1			0.0000	0.1630	0.2066	10	10	10
14	OLS M2			0.0000	0.1584	0.2024	3	3	3
15	OLS M3			**0.0000**	**0.1584**	**0.2024**	4	2	3
16	TOBIT M1			−0.0011	0.1627	0.2066	9	11	10
17	TOBIT M2			−0.0012	0.1579	0.2024	2	4	3
18	TOBIT M3			−0.0012	0.1579	0.2024	1	5	3

.: the analysis is not converge

### Mapping on SF-6D

Table 6 shows the performance of three regression methods for mapping the PHQ-8 to the SF-6D utility scores. Among the three regression methods and 18 model specifications, beta mixture regression method with two components without truncation and probability mass at full health (1) was found to be the most parsimonious prediction model for the SF-6D utility scores. It produced the smallest average ranking of MAE (0.0519) and RMSE (0.0683). In this regression model, PHQ-8 total scores and age were significantly and negatively associated with the SF-6D utility scores in both components. Meanwhile, the female gender was significantly and negatively associated with the SF-6D utility scores in the first component (Supplementary Table 4).

**Table 6:** Model performance of three regression methods for mapping the PHQ-8 to the SF-6D utility scores

No	Mapping method	Number of components and truncation	Specification	ME	MAE	RMSE	MAE rank	RMSE rank	ARV
1	BETAMIX M1a	1 component without truncation	Probability mass at full health	0.0008	0.0587	0.0746	10	9	9.2
2	BETAMIX M1b	2 components without truncation	Probability mass at full health	0.0060	0.0575	0.0749	9	10	9.2
3	BETAMIX M1c	2 components with truncation	Probability mass at full health	N/A	N/A	N/A	N/A	N/A	N/A
4	BETAMIX M1d	2 components with truncation	Probability mass at full health and truncation point	N/A	N/A	N/A	N/A	N/A	N/A
5	BETAMIX M2a	1 component without truncation	Probability mass at full health	0.0013	0.0539	0.0695	6	6	6
6	BETAMIX M2b	2 components without truncation	Probability mass at full health	0.0055	0.0527	0.0693	2	5	3.5
7	BETAMIX M2c	2 components with truncation	Probability mass at full health	N/A	N/A	N/A	N/A	N/A	N/A
8	BETAMIX M2d	2 components with truncation	Probability mass at full health and truncation point	N/A	N/A	N/A	N/A	N/A	N/A
9	BETAMIX M3a	1 component without truncation	Probability mass at full health	0.0020	0.0533	0.0683	5	2	3.5
10	BETAMIX M3b	2 components without truncation	Probability mass at full health	**0.0057**	**0.0519**	**0.0683**	1	1	1
11	BETAMIX M3c	2 components with truncation	Probability mass at full health	N/A	N/A	N/A	N/A	N/A	N/A
12	BETAMIX M3d	2 components with truncation	Probability mass at full health and truncation point	N/A	N/A	N/A	N/A	N/A	N/A
13	OLS M1			0.0000	0.0587	0.0754	11	11	11
14	OLS M2			0.0000	0.0545	0.0710	7	7	7
15	OLS M3			0.0000	0.0532	0.0686	3	3	3
16	TOBIT M1			0.0000	0.0587	0.0754	12	12	12
17	TOBIT M2			0.0000	0.0545	0.0710	8	8	8
18	TOBIT M3			0.0000	0.0532	0.0686	4	4	4

*N/A* Not available due to no values at the upper boundary of full health

## Discussion

The current study is among the few that have been conducted to map PHQ-8 scores on four common utility scores, the EQ-5D-3L, EQ-5D-5L, HUI3, and SF-6D, among people with depression in a multiethnic Asian population. In the current study, three different regression methods with 18 model specifications were explored to find the most parsimonious prediction model to develop mapping functions from the PHQ-8. The findings provide evidence that different predictive models should be used for mapping EQ-5D-3L, EQ-5D-5L, HUI3, and SF-6D in our sample. Our analyses showed that both versions of the EQ-5D utility scores were best predicted by the beta mixture regression model, consistently reported in other studies [20–22]. Our mapping algorithm for the HUI3 was best predicted by the ordinary least square model with minimal MSE and MAE values. We found PHQ-8 total scores, PHQ-squared scores, as well as age and gender to play a significant role in mapping the utility scores in the expected direction in the depression sample. For example, our findings show that the PHQ-8 total scores were significantly and negatively associated with the HUI3 and SF6D utility scores, while the quadratic term of the PHQ-8 total scores (i.e., PHQ-squared) was significantly and negatively associated with both the EQ-5D-3L and EQ-5D-5L. This reflects significant concave relationships between PHQ-8 total scores and EQ-5D utility scores. Our findings also show that age was significantly and negatively associated with the EQ-5D-5L, HUI3, and SF-6D utility scores, while the female gender was significantly and negatively associated with the SF-6D utility scores. These findings suggest that it is important to include patient’s key demographic characteristics such as age and gender to map the utility scores in the depression sample. It is important to note that the primary intention of the study is to develop a mapping function that best predicts utility scores derived from EQ-5D-3L, EQ-5D-5L, HUI3, and SF-6D, so whether the regression coefficients are statistically significant is of secondary consideration [24]. In the current study, model selection was primarily determined by the MAE and MSE. In order to avoid bias, the choice of the best model was based on the average ranking of both indices instead of focusing exclusively on one fit index.

Several limitations should be acknowledged in the current study. Firstly, the utility values for EQ-5D-5L were based on a crosswalk project that maps EQ-5D-5L utility scores from the EQ-5D-3L. Secondly, due to the small sample size, we were unable to test whether the model works equally well in sub-samples of the overall sample. However, a recent guideline by the ISPOR Good Practice for Outcomes Research Task Force has not recommended splitting the sample to validate results on part of the sample [25]. Hence, further validation of the current mapping findings using an external dataset is recommended. Nonetheless, to our knowledge, this is the first study to use beta mixture regression model against the Tobit and linear regression methods to map the PHQ-8 scale onto widely used generic preference-based measures specifically for depression patients.

In conclusion, we have generated the algorithm for converting PHQ-8 scores into utility scores that are easily applicable in the clinical setting when the EQ-5D-3L, EQ-5D-5L, HUI3, and SF-6D data are not available. The current study provides necessary details to clinicians and researchers on mapping algorithms that can be used in economic evaluations among patients with depression.

## Supplementary Information

**Additional file 1.**

## Data Availability

Data are available upon reasonable request. Raw data from this study are currently not publicly available but can be made available upon reasonable request from the corresponding author.
