# Supplementary material for: Mapping the PHQ-8 to EQ-5D, HUI3 and SF6D in patients with depression
Source: BMC Psychiatry. 2021 Sep 13;21:451. doi: 10.1186/s12888-021-03463-0 (PMC8438835; doi:10.1186/s12888-021-03463-0)
Supplement: Supplementary file 1 — Additional file 1. [file 12888_2021_3463_MOESM1_ESM.docx]

**Supplementary Tables**

Supplementary Table 1

Final regression method for mapping PHQ-8 to the EQ-5D-3L

| eq5d | Coef. | P value | [95% Conf. | Interval] |
| --- | --- | --- | --- | --- |
|  |  |  |  |  |
| C1_mu |  |  |  |  |
| PHQ | 0.008458 | 0.881 | -0.10238 | 0.119301 |
| PHQ squared | -0.00549 | 0.008 | -0.00957 | -0.00141 |
| Age | -0.01394 | 0.067 | -0.02885 | 0.000964 |
| Female | 0.115741 | 0.459 | -0.19035 | 0.42183 |
| Constant | 2.609336 | <0.001 | 1.567338 | 3.651333 |
|  |  |  |  |  |
| C1_lnphi |  |  |  |  |
| Constant | 1.205062 | <0.001 | 0.919409 | 1.490715 |
|  |  |  |  |  |
| C2_mu |  |  |  |  |
| PHQ | 0.465364 | 0.548 | -1.05407 | 1.984795 |
| PHQ squared | -0.01989 | 0.419 | -0.06815 | 0.028375 |
| Age | -0.01291 | 0.798 | -0.11164 | 0.085825 |
| Female | 0.120641 | 0.901 | -1.78573 | 2.027011 |
| Constant | -1.52292 | 0.799 | -13.2707 | 10.22487 |
|  |  |  |  |  |
| C2_lnphi |  |  |  |  |
| Constant | -0.92327 | 0.123 | -2.09569 | 0.249152 |
|  |  |  |  |  |
| Prob_C1 |  |  |  |  |
| Constant | 2.839245 | <0.001 | 1.398788 | 4.279702 |
|  |  |  |  |  |
| PM_ub |  |  |  |  |
| PHQ | -0.64928 | <0.001 | -0.91326 | -0.3853 |
| PHQ squared | 0.018048 | 0.005 | 0.005515 | 0.030582 |
| Age | -0.0254 | 0.281 | -0.0716 | 0.020799 |
| Female | -0.17705 | 0.722 | -1.15124 | 0.797149 |
| Constant | 2.677106 | 0.015 | 0.529058 | 4.825153 |
|  |  |  |  |  |
| PM_tb |  |  |  |  |
| PHQ | -0.47662 | 0.159 | -1.14015 | 0.186912 |
| PHQ squared | 0.002683 | 0.922 | -0.05125 | 0.05662 |
| Age | 0.021052 | 0.606 | -0.05897 | 0.10107 |
| Female | 1.996548 | 0.085 | -0.27307 | 4.266171 |
| Constant | -2.3773 | 0.272 | -6.62206 | 1.867466 |
|  |  |  |  |  |
| C1_phi | 3.336966 |  | 2.507807 | 4.440269 |
| C2_phi | 0.397219 |  | 0.122986 | 1.282937 |
| pi1 | 0.94476 |  | 0.801992 | 0.986342 |
| pi2 | 0.05524 |  | 0.013658 | 0.198009 |

Supplementary Table 2

Final regression method for mapping PHQ-8 to the EQ-5D-5L

| eq5d5l | Coef. | P value | [95% Conf. | Interval] |
| --- | --- | --- | --- | --- |
|  |  |  |  |  |
| C1_mu |  |  |  |  |
| PHQ | -0.08069 | 0.698 | -0.4878 | 0.326408 |
| PHQ squared | -0.00447 | 0.57 | -0.01991 | 0.010964 |
| Age | -0.05567 | 0.05 | -0.11134 | -1.05E-06 |
| Female | 1.219305 | 0.06 | -0.05066 | 2.489272 |
| Constant | 3.190162 | 0.056 | -0.08755 | 6.467876 |
|  |  |  |  |  |
| C1_lnphi |  |  |  |  |
| _cons | 1.226452 | 0.002 | 0.444723 | 2.00818 |
|  |  |  |  |  |
| C2_mu |  |  |  |  |
| PHQ | 0.02692 | 0.498 | -0.0509 | 0.104737 |
| PHQ squared | -0.00394 | 0.007 | -0.00683 | -0.00106 |
| Age | -0.00611 | 0.346 | -0.01881 | 0.006597 |
| Female | -0.09023 | 0.486 | -0.3438 | 0.163336 |
| Constant | 2.078374 | <0.001 | 1.366825 | 2.789922 |
|  |  |  |  |  |
| C2_lnphi |  |  |  |  |
| _cons | 2.225145 | <0.001 | 1.848281 | 2.602009 |
|  |  |  |  |  |
| Prob_C1 |  |  |  |  |
| _cons | -1.93758 | 0.001 | -3.07003 | -0.80512 |
|  |  |  |  |  |
| PM_ub |  |  |  |  |
| PHQ | 0.470157 | 0.222 | -0.28515 | 1.225466 |
| PHQ squared | -0.11835 | 0.027 | -0.22332 | -0.01338 |
| Age | -0.07526 | 0.02 | -0.13879 | -0.01173 |
| Female | -1.08338 | 0.1 | -2.37425 | 0.207478 |
| Constant | 3.045425 | 0.031 | 0.284652 | 5.806198 |
|  |  |  |  |  |
| PM_tb |  |  |  |  |
| PHQ | 0.062733 | 0.629 | -0.19194 | 0.31741 |
| PHQ squared | -0.01213 | 0.088 | -0.02609 | 0.001822 |
| Age | -0.00324 | 0.86 | -0.03914 | 0.032661 |
| Female | 0.327139 | 0.417 | -0.46205 | 1.116329 |
| Constant | -0.85054 | 0.391 | -2.79365 | 1.092564 |
|  |  |  |  |  |
| C1_phi | 3.409111 |  | 1.560058 | 7.449748 |
| C2_phi | 9.254828 |  | 6.348899 | 13.49082 |
| pi1 | 0.125915 |  | 0.044361 | 0.308931 |
| pi2 | 0.874086 |  | 0.691069 | 0.95564 |

Supplementary Table 3

Final regression method for mapping PHQ-8 to the HUI3

| hui3 | Coef. | P value | [95% Conf. | Interval] |
| --- | --- | --- | --- | --- |
|  |  |  |  |  |
| PHQ | -0.03961 | <0.001 | -0.05379 | -0.02542 |
| PHQ squared | -2.8E-05 | 0.925 | -0.00061 | 0.000558 |
| Age | -0.00339 | 0.007 | -0.00583 | -0.00095 |
| Female | -0.02521 | 0.35 | -0.07824 | 0.027818 |
| Constant | 1.057726 | <0.001 | 0.934002 | 1.18145 |

Supplementary Table 4

Final regression method for mapping PHQ-8 to the SF-6D

| sf6d | Coef. | P value | [95% Conf. | Interval] |
| --- | --- | --- | --- | --- |
|  |  |  |  |  |
| C1_mu |  |  |  |  |
| PHQ | -0.083 | <0.001 | -0.12108 | -0.04491 |
| PHQ squared | 0.000962 | 0.212 | -0.00055 | 0.002473 |
| Age | -0.01117 | <0.001 | -0.01743 | -0.00491 |
| Female | -0.17492 | 0.01 | -0.30794 | -0.04191 |
| Constant | 0.779218 | <0.001 | 0.437513 | 1.120924 |
|  |  |  |  |  |
| C1_lnphi |  |  |  |  |
| _cons | 3.632685 | <0.001 | 3.305588 | 3.959781 |
|  |  |  |  |  |
| C2_mu |  |  |  |  |
| PHQ | -0.23379 | <0.001 | -0.33924 | -0.12834 |
| PHQ squared | 0.0034 | 0.16 | -0.00134 | 0.00814 |
| Age | -0.04281 | <0.001 | -0.06634 | -0.01927 |
| Female | 0.316254 | 0.163 | -0.12795 | 0.760452 |
| Constant | 2.824478 | <0.001 | 1.796259 | 3.852698 |
|  |  |  |  |  |
| C2_lnphi |  |  |  |  |
| _cons | 1.893264 | <0.001 | 1.519144 | 2.267383 |
|  |  |  |  |  |
| Prob_C1 |  |  |  |  |
| _cons | 0.917262 | 0.001 | 0.363721 | 1.470802 |
|  |  |  |  |  |
| PM_ub |  |  |  |  |
| PHQ | 71.6895 | 0.998 | -53947.3 | 54090.62 |
| PHQ squared | -17.8681 | 0.998 | -12533.1 | 12497.31 |
| Age | 0.041873 | 0.736 | -0.20168 | 0.285428 |
| Female | . | . | . | . |
| Constant | -74.9152 | 0.998 | -60434.5 | 60284.63 |
|  |  |  |  |  |
| C1_phi | 37.8142 |  | 27.26456 | 52.44586 |
| C2_phi | 6.641008 |  | 4.568313 | 9.654107 |
| pi1 | 0.714484 |  | 0.589941 | 0.813179 |
| pi2 | 0.285516 |  | 0.186821 | 0.410059 |
